# Acid-Sensing Ion Channels in Glial Cells

**DOI:** 10.3390/membranes12020119

**Published:** 2022-01-20

**Authors:** Victoria Cegielski, Rohan Chakrabarty, Shinghua Ding, Michael J. Wacker, Paula Monaghan-Nichols, Xiang-Ping Chu

**Affiliations:** 1 Department of Biomedical Sciences, School of Medicine, University of Missouri-Kansas City, Kansas City, MO 64108, USA; vicbmy@umkc.edu (V.C.); rohan.chakrabarty@umkc.edu (R.C.); wackerm@umkc.edu (M.J.W.); nicholsap@umkc.edu (P.M.-N.); 2 Department of Biomedical, Biological and Chemical Engineering, University of Missouri-Columbia, Columbia, MO 65211, USA; dings@missouri.edu; 3 Dalton Cardiovascular Research Center, University of Missouri-Columbia, Columbia, MO 65211, USA

**Keywords:** acid-sensing ion channels, glial cells, astrocyte, microglia, oligodendrocyte, expression, function

## Abstract

Acid-sensing ion channels (ASICs) are proton-gated cation channels and key mediators of responses to neuronal injury. ASICs exhibit unique patterns of distribution in the brain, with high expression in neurons and low expression in glial cells. While there has been a lot of focus on ASIC in neurons, less is known about the roles of ASICs in glial cells. ASIC1a is expressed in astrocytes and might contribute to synaptic transmission and long-term potentiation. In oligodendrocytes, constitutive activation of ASIC1a participates in demyelinating diseases. ASIC1a, ASIC2a, and ASIC3, found in microglial cells, could mediate the inflammatory response. Under pathological conditions, ASIC dysregulation in glial cells can contribute to disease states. For example, activation of astrocytic ASIC1a may worsen neurodegeneration and glioma staging, activation of microglial ASIC1a and ASIC2a may perpetuate ischemia and inflammation, while oligodendrocytic ASIC1a might be involved in multiple sclerosis. This review concentrates on the unique ASIC components in each of the glial cells and integrates these glial-specific ASICs with their physiological and pathological conditions. Such knowledge provides promising evidence for targeting of ASICs in individual glial cells as a therapeutic strategy for a diverse range of conditions.

## 1. Introduction

Acid-sensing ion channels (ASICs) are proton-gated cation channels found extensively in the nervous system [1,2]. At least four genes (*ACCN 1 to 4*) have been cloned so far and are categorized as *ASIC1-4*, with two splice variants identified in ASIC1 and ASIC2 [3,4]. ASICs have more than five-hundred amino acids and consist of two hydrophobic cysteine-rich extracellular loops with intracellular N and C terminals [5,6]. The crystal structure of ASIC1a reveals three subunits forming functional ion channels with multitrimers, heteromers, and homomers [7,8,9]. Protons or non-proton ligands activate ASICs rendering them permeable to Na^+^ ions mostly, thus the ASICs belong to the degenerin/epithelial sodium channel (DEG/ENaC) family [10,11]. Homomeric ASIC1a is unique in that it is also calcium permeable [12,13]. ASICs also exhibit unique half activation pH values (pH_50_), with functional homomeric ASIC1a, 1b, 2a, and 3 activated at pHs of 6.5, 5.9, 4.4, and 6.6, respectively [14].

Prior reviews have not gathered ASIC information for each of the glial cell types nor discussed how the glial ASICs relate to specific physiology and pathology. Furthermore, most reviews concentrate on ASICs in neurons, while very few have covered their role in glial cells. ASICs are widely expressed in neurons and are less expressed in glial cells throughout the nervous system [15,16]. ASIC1a and ASIC2 are highly expressed in both the central and peripheral nervous system [17,18]. On the other hand, ASIC1b and ASIC3 are primarily expressed in the peripheral sensory neuron [19,20,21]. Protons are ligands for ASICs and have been shown to act as neurotransmitters in the brain [11,22]. Other non-proton ligands have also been shown to activate these channels at a physiological pH (e.g., pH 7.4) including the exogenous agent, 2-guanidine-4-methylquinazoline (GMQ), which has been found to activate ASIC3 [23,24], and the coral snake toxin, MitTx, which activates ASIC1 [25].

In light of the pivotal role that ASICs play in several physiological and pathological processes [26,27], select pharmacological blockers of ASICs have been identified and widely used for both research and clinical purposes [28,29,30]. Amiloride, an ENaC blocker, is a non-selective and widely used inhibitor of ASICs [1], whereas A-317567 [31] and α-dendrotoxin [32] have been reported to block the ASIC currents in dorsal root ganglion neurons. The spider toxin, psalmotoxin-1 (PcTx1), is a selective ASIC1a inhibitor [33,34] that has profound neuroprotective effects against brain ischemia [13], whereas the sea anemone agent, APETx2, is a selective ASIC3 inhibitor that has been shown to modulate pain [35,36]. Mambagin, a toxin isolated from black mamba snakes, has been shown to strongly inhibit ASIC1 channels [37,38]. The availability of these select inhibitors has facilitated the dissection of their role in distinct cellular processes [39].

### 1.1. Importance of Glial Cells in Neuronal Function

There are three types of glial cells in the central nervous system (CNS): astrocytes, microglia, and oligodendrocytes [40]. Each of these cell types has a specific role which helps the brain maintain normal physiological function [41,42,43,44,45,46,47,48,49,50]. Astrocytes regulate synaptic transmission [51,52,53], microglia are the primary executors of phagocytosis [54,55], and oligodendrocytes enhance synaptic efficiency by forming myelin sheaths [56,57]. It is worthwhile mentioning that while we discuss the functions of glial cells under conditions of their ablation, glial cell function is highly complex and there exists varied, plastic phenotypes [41,42,43,44,45,46,47,48,49,50,51,52,53,54,55,56,57,58].

Astrocytes critically regulate neurotransmission and neuronal function throughout the CNS [42,43,58]. These cells express high quantities of transporters that are necessary for reuptake of the excitatory amino acid, glutamate [52]. Glutamate excitation is important in regulation of neuronal transmission and coordination of normal motor activity [59]. When astrocytes are nonfunctional and glutamate persists unopposed, inappropriate synaptic regulation and transmission can lead to axonal degeneration and subsequent limb paralysis. Along the same lines, astrocytic ablation results in higher levels of dangerous reactive oxidative species (ROS), indicating that astrocytes play an important role in maintaining redox homeostasis for survival [60]. Astrocytes are also important in regulation of extracellular K^+^ and alterations of blood flow [61,62].

Microglia are essential in maintaining proper brain function through environment surveillance and clearance [44,45,46,47]. Although microglia repopulate much quicker than astrocytes [63,64], loss of the microglial cell line causes transient effects on spatial memory [65] and learning behavior [66]. Microglia perform the critical roles of monitoring and phagocytosis in the brain, allowing for proper immune response and effective debris clearance [67]. Constant monitoring and removal of problem materials by these cells promotes a healthy CNS that functions successfully.

Oligodendrocytes play an important role in promoting effective myelin performance [48,49,50]. More importantly, oligodendrocytes and axons have a complex reciprocal relationship, and oligodendrocytes not only myelinate axons but subsequently shape axonal structure and integrity by providing trophic factors and metabolic support for neurons [49]. Most studies of oligodendrocyte ablation report demyelination as the primary consequence, as well as decreased maintenance and shaping of axonal structure and integrity [68,69,70,71]. Secondary findings vary with reports of subsequent remyelination and motor impairment [68], gait disturbances and tremors [68,69], and neuropathic pain [70]. Therefore, oligodendrocytes are necessary for myelin formation and function, ultimately promoting efficient CNS performance.

### 1.2. Glial Cell Function in Pathological Conditions

Under abnormal conditions, glial cells exhibit different behaviors which contribute to the appearance of pathology [53,54,55]. Among astrocytes in the glial fibrillary acidic protein (GFAP)-herpes simplex virus-thymidine kinase (HSV-TK) mouse line, 95% astrocytic deletion in a spinal cord with stab wound injuries resulted in increased tissue necrosis and higher levels of inflammation [72]. Similar destructive tissue findings were reported in studies that induced astrocytic disruption after experimental autoimmune encephalitis (EAE) to mimic chronic adaptive immune inflammation [73] and traumatic brain injury to the cortex to simulate an acute local inflammatory event [74]. These studies suggest that astrocytes are principal mediators for maintaining brain homeostasis in pathological circumstances. Astrocytes also ensure the efficiency of repair mechanisms on injured areas and prevent the inflammatory response from spreading to locations outside the injury itself [74].

In microglial cells exposed to pathological conditions, contrary effects are observed. In an experiment with over 90% CD11b-HSV-TK microglia ablation during EAE conditions, inflammatory effects were repressed [75]. This indicates that under inflammatory pathological conditions, microglia perpetuate disease-state conditions by increasing inflammatory effects. On the other hand, in the same mouse line of CD11b-HSV-TK, 75% microglial ablation in mouse middle cerebral artery occlusion (MCAO) model resulted in a larger area of infarct and more pronounced neuronal death [76]. Under these ischemic conditions, microglia diminish the extent of injury and reveal a protective function against brain ischemia. Therefore, microglia demonstrate different effects in different pathological states; in inflammatory conditions, microglia tend to perpetuate damage, whereas in acute ischemic conditions, microglia alleviate the extent of injury.

Deletion of oligodendrocytes under specific pathological conditions has not been experimentally performed yet. As discussed earlier, however, many studies have ablated oligodendrocytes under healthy physiological conditions. All these studies report similar results of demyelination and disintegrated axonal support [68,69,70,71], with varying secondary effects.

ASICs are expressed in glial cells [67], indicating that they could play certain roles in physiological and pathological conditions. In this review, we concentrate on the unique ASIC components in each of the glial cells and integrate these glial-specific ASICs with their physiological and pathological conditions. Understanding the prevalence and function of ASICs is an important step toward potential breakthrough therapies, and targeting of ASICs in individual glial cells could serve as an ideal therapeutic to delay neurodegeneration, decrease ischemic brain injury, and prevent progression of glial brain tumors.

## 2. ASICs in Astrocytes

ASIC1, ASIC2, and ASIC3 have been detected in the astrocytes [77]. ASIC1 contributes to rapid depolarization of astrocytes, whereas ASIC2 and ASIC3 generate sustained currents and thus are slowly depolarizing [77]. Of these, ASIC1a is the most prevalent ASIC in human astrocytes [78]. ASIC1a colocalizes with other ion channel subunits, including α and γ ENaC, to form a channel on the plasma membrane of astrocytes and induce depolarization upon stimulation [79]. ASIC1a physiologically functions by allowing the influx of Na^+^ and Ca^2+^ ions when pH levels drop below 7.0 [78]. In astrocytes, such depolarization and signaling might be fundamental to the aforementioned functions of motor coordination [52,59,80], ROS mediation [60,81], and blood flow regulation [61,82]. However, the exact role of ASIC1a in astrocytes is not certain, and future studies would need to directly focus on this using astrocyte-specific genetic methods in order to determine its significance in physiology and pathology. Furthermore, the specific role of ASIC2 and ASIC3 in astrocytes is yet to be elucidated.

One feature that distinguishes functional ASIC1a from other ASIC components is its unique permeability to Ca^2+^, in addition to Na^+^ [12,13]. Calcium influx occurs during activation of astrocytic ASIC1a, as well as trafficking ASIC1a to the cell membrane under acidic extracellular conditions [78]. Intracellular Ca^2+^ plays an important role in long-term potentiation (LTP), which is critical for synaptic plasticity that contributes to learning and memory by activation of ASIC1a in neurons [83,84,85]. Astrocytic ASIC1a might contribute to synaptic plasticity such as LTP and long-term depression (LTD). This can definitively be confirmed in future studies since ASIC1a knockout (KO) mice are available [83].

ASICs are involved in neurodegenerative diseases [26,86,87]. The following experiments highlight the role of ASICs in exacerbating disease states. Using EAE as the experimental mouse model of multiple sclerosis (MS), the role of ASIC1 in this pathological state was examined [88]. It was found that ASIC1 KO mice had less axonal degeneration than their wild-type (WT) mice counterparts, indicating that ASIC1 progresses the degenerative process of MS [88]. This was very similar to findings in mice induced by 1-methyl-4-phenyl-1,2,3,6-tetrahydropyridine, a chemical model of Parkinson’s disease (PD) [89]. PD is characterized by loss of dopaminergic neurons in the substantia nigra, where ASIC1a components are also located [89]. When ASIC1a was inhibited by its very effect inhibitor PcTx1, neurons were protected from degeneration [89]. In transgenic mice with the mutated ataxin-1 gene, a phenotype corresponding to spinocerebellar type 1 ataxia was observed, ASIC1 KO mice presented with improved motor function under this phenotype [90]. ASIC1 KO mice also upregulate the ubiquitin-proteasome system, which has been implicated in decreased huntingtin-polyglutamine accumulation [91]. The improved function of mice with deletion of ASIC1 or inhibition of ASICs support the idea of protective effects under ASIC1 inhibition or disruption. While there is more literature focusing on ASIC1 in neurons, it is important to note that ASIC1 has also been discovered in astrocytes. This puts forth the question of the exact role that astrocytic ASIC1 may play in conditions such as neurodegeneration. It is possible that ASIC1 in astrocytes is the primary channel disrupting homeostasis in these pathological conditions, but ASIC1 in neurons, oligodendrocytes, and microglia could also shape axonal structure and integrity. Therefore, astrocyte-specific deletion using conditional KO mice by crossing astrocyte-specific Cre driver mice such as GLAST-CreERT2, GFAP-CreERT2, and ALDH1L1-CreERT2 lines with floxed ASIC1 mice would be necessary in order to distinguish the specific function of ASIC1 in astrocytes.

In a mouse MCAO model, ASIC1a demonstrated a role in ischemic brain injury due to its calcium-dependent or calcium-independent mechanisms [13,92]. Activation of ASIC1a by acidosis, as a consequence of ischemic brain injury, leads to elevated intracellular calcium which contributes to pathological distress [13]. Additionally, the acidic environment created by strokes also causes ASIC1a to couple with an enzyme, receptor-interacting serine/threonine-protein kinase 1, triggering neuronal death through necroptosis [93,94]. Increased protease activity as a result of stroke has been implicated in increased ASIC1a activation which further contributes to ischemic brain injury [95]. Given these findings, it follows that when ASIC1a is inhibited under ischemic conditions, neuroprotection is observed [13,96]. Future studies can concentrate on ASIC1a in astrocytes to localize specific cellular changes contributing to brain ischemia.

Finally, a role for ASICs in brain tumors has been established. ASIC1a is highly expressed in malignant gliomas [97,98] and selective inhibition of ASIC1a by PcTx1 leads to a decrease in the rate of glioma cell growth [99] and significant inhibition of glioma cell migration [79,100]. Interestingly, ASIC2 in astrocytes inhibits the growth of gliomas [101]. When ASIC2 is not expressed, specific negative effectors requiring this channel are nonfunctional. This causes ASIC1 action to be unopposed, and leads to a basally-active cation current which characterizes glioma cells [101]. The results of these ASIC experiments demonstrate the integral role that ASIC1a has in accelerating glioma. In addition to neoplastic growth, ASIC1a activation is also a likely contributing factor to the high rates of epileptic seizures seen in glioma patients. This seizure-prone behavior is a consequence of extracellular acidosis which, in the absence of any neuronal signaling, induces ASIC1a depolarization in glioma cells and triggers electrical activity in brain circuits [102]. Furthermore, ASIC1a in gliomas induces electrical currents that promote cell proliferation, perpetuating the tumor prognosis [102]. This is confirmed by the fact that glioma proliferation and migration is inhibited by the ASIC1a inhibitor toxin, PcTx1 [79,97,98], as well as ASIC2 [101]. Therefore, ASIC1a in gliomas has widespread effects, through both malignancy of the cancer itself, as well as neurological side effects. Research focused on ASIC1a in astrocytes of gliomas may provide a direction in determining cellular targets to prevent cancer progression.

## 3. ASICs in Microglial Cells

ASIC1a, 2a and 3 are expressed in microglial cells [103]. Another ion channel, voltage-gated proton channel (H_V_) subtype 1, is prominently expressed in microglial cells and plays a role in free radical generation that contributes to ischemic brain injury [104]. Recent studies have confirmed that H_V_1 is also involved in electrical signaling by activating ASIC1a in microglial cells, leading to further proton influx of Na^+^ and Ca^2+^ [105].

ASIC1a and ASIC2a contribute to the physiological function of microglial cells [87,103]. As was noted in the astrocyte section, ASIC1 is sensitive to small drops in pH; its threshold is around 7.0 [14,106]. ASIC2a, on the other hand, is not as sensitive to drops in pH and is only activated under much more acidic conditions, with a pH threshold of 5.5 for activation of ASIC2a homomers [14]. One of the physiological roles of ASIC2a is to increase ASIC1 co-localization to dendritic spines, facilitating normal neuronal function [107,108]. Without ASIC2a expression, there is less ASIC1 trafficking to cell membranes [107,108]. ASIC3 is primarily involved with pain modulation [15,21,109,110,111]. It has a pH threshold close to that of ASIC1 [4,112,113], and can be activated for experimental purposes by the non-proton ligand GMQ [23,24]. When ASIC3 is highly expressed in microglial cells due to low pH conditions, hyperalgesia to various insults is observed [114]. Furthermore, when ASIC3 is genetically deleted or inhibited by the peptide toxin, APETx2, fatigue-induced hyperalgesia is diminished [114].

The primary function of microglia is their role in environment surveillance and phagocytosis [63,115]. As microglia age, their execution in debris removal decreases; this dysfunction plays a major role in plaque formation. A_β_ plaque accumulation is characteristic of Alzheimer’s disease (AD) [116]. In addition to decreased efficacy in phagocytosis, aged microglia produce storms of inflammatory cytokines and ROS that create a positive feedback loop contributing to continued plaque formation and microglial dysfunction [116]. In particular, ASIC1 and ASIC2a are the most prevalent inflammatory respondents in microglia. This was demonstrated by the upregulation of ASIC1 and ASIC2a when stimulated by LPS, an inflammatory inducer [61,103]. Under LPS stimulation, when the nonspecific ASIC inhibitor, amiloride, or the selective ASIC1a inhibitor, PcTx1, were added, depolarization was prevented and there was reduced expression of inflammatory cytokines that activate enzymes such as nitric oxide (NO) synthase and cyclooxygenase 2 [103]. Such findings demonstrate how ASIC1 and ASIC2a in microglial cells contribute to cytokine storms. Consequently, when inflammation is overstimulated and causes overaccumulation of inflammatory cytokines and cellular debris, cellular senescence and neurodegeneration pursue [117].

Our recent study focusing on ASIC2 pathogenesis in ischemic brain injury found that in hippocampal, cortical, and striatal neurons, deletion of ASIC2 reduced surface ASIC1a levels, acid-activated current density, and acidosis/ischemia-induced neuronal injury [108]. We also found that, in cerebellar neurons with much less ASIC2 expression, ASIC2 deletion had no effect on surface ASIC1a level, acid-activated current density, or acidosis/ischemia-induced neuronal injury, suggesting that heteromeric ASIC1a/2 channels in certain brain regions are likely to contribute to ischemic brain injury [108]. With the established connection between ASIC1a and ischemic brain injury, these findings open the door for future research surrounding neuromodulation of ASIC2 to help mitigate neuronal injury after stroke. This could play an important role in targeted therapy due to the fact that there is a region-specific difference in the pathophysiology of ASIC2. Due to ASIC2 KO mice used in these studies, one possibility is that microglial ASIC2 is involved in this process. However, future studies need to explore whether microglial ASIC2 is directly involved in the ischemic brain injury.

Expression of ASIC3 in response to stimuli induces primary and secondary hyperalgesia [87]. Thus, pain associated with primary inflammation or inflammation induced by microglial dysfunction (as seen in AD) could potentially be attributed to ASIC3. Future research concentrated on the role of ASIC3 in microglial cells could provide evidence for therapeutic targeting against this cellular channel to diminish pain associated with certain neurological conditions.

## 4. ASICs in Oligodendrocytes

Using imaging and immunofluorescence techniques, it has been determined that ASIC1a, ASIC2a, and ASIC4 mRNAs are expressed in oligodendrocyte lineage cells (OLC) [118]. When oligodendrocytes are treated with PcTx1, a selective inhibitor for ASIC1a, electrical currents are almost completely blocked as well, indicating a high expression of ASIC1a in OLC [118]. This proves that ASIC1a is found extensively in oligodendrocytes, and thus plays a substantial role in white matter function.

Oligodendrocytes serve primarily to form myelin sheaths which enhance electrical conduction of synaptic transmissions in the brain [57]. NG2-glia, also known as oligodendrocyte precursor cells (OPC), function to give rise to mature oligodendrocytes that then allow for myelin sheath formation [119]. OPC also forms tight homeostatic networks that maintain cell numbers. Thus, they are very effective for repopulation [52]. ASIC1a is the predominant ASIC component found in OPC and mature oligodendrocytes [119]. ASIC1a plays a significant role in the signaling pathways of OLC [118], which ultimately leads to myelin production and rapid saltatory conduction.

Oligodendrocyte dysfunction is implicated in several disease processes, most notably multiple sclerosis (MS) [120]. MS is characterized as an autoimmune disease resulting in progressive demyelination of CNS axons, ultimately leading to severe neurological pathology [121]. One proposed mechanism relates to the propensity for ASIC1 oligodendrocytes to be highly susceptible to calcium overload [122,123]. Recent studies show that ASIC2 also contributes to MS [124]. Using the EAE model in ASIC1 and ASIC2 KO mice, a significant reduction in clinical scoring was found not only in ASIC1 KO mice, but also in ASIC2 KO mice at days 20–23 after immunization [124]. The underlying mechanism of ASIC1a involved in MS might be due to activation of ASIC1a resulting in overload of intracellular Ca^2+^ in the EAE model [123]. Modulation of ASIC1a surface expression by deletion of ASIC2 might also contribute to MS [108], but this should be investigated in future studies.

OPC plays an important role in chronic conditions such as obesity and cancer [125,126]. OPC makes contact with leptin receptors on dendritic processes, which helps to regulate the electrical response of leptin in the body [125]. Since ASIC1a is prominently found in OPC [119], it can be postulated that ASIC1a holds a significant role in leptin signaling. When OPC is destroyed or ASIC1 in OPC are not functioning properly, obesity risk is hypothesized to increase [125]. Furthermore, OPC proteins are highly expressed in gliomas and are shown to promote tumor origination and progression [126]. Again, since ASIC1a is predominantly found in OPC, it can be predicted that this channel plays a specific role in OPC function. A direct focus on this in future studies would be necessary to confirm.

## 5. Perspective

ASICs are expressed in glial cells. In this review, we discussed the integral roles that ASICs play in glial cell physiology and pathology, and how each unique ASIC component contributes to the specified function of each glial cell type. While lots of information exists on the topic, there are still many knowledge gaps within the literature. This section aims to summarize the major findings of the review and provide guidance for future research in the field.

### 5.1. Astrocytic ASICs

Astrocytes play major roles in support and regulation of physiological and pathological states [42,53,58]. While multiple ASICs exist within astrocytes, ASIC1 plays a predominant role in both healthy and disease-state conditions [78]. Physiologically, ASIC1a plays a unique role by contributing to motor coordination, homeostasis, and LTP in learning and memory through depolarization and Ca^2+^ influx [84,85,106]. Pathologically, ASIC1a demonstrates a specific contribution to the progression of various disease-state conditions. Thus, when ASIC1a is inhibited, protective effects in neurodegenerative diseases, ischemic conditions, and brain tumor are observed [13,88,89,90,126].

Despite these general findings, no studies directly focus on this relationship between ASICs in astrocytes and specific physiological or pathological outcomes. For example, there has been a lot of evidence that inhibition of ASIC1a or deletion of ASIC1a gene in neurons leads to protection during pathological conditions [26,27], but no research has specifically targeted astrocytic ASIC1a and explored its effects. Therefore, future research in the field should target the ASIC1a component of astrocytes using astrocyte-specific conditional KO mice to garner a more explicit understanding of its biological role. This could ultimately lead to the development of targeted astrocytic ASIC1a therapies for serious neurological diseases. For instance, stroke and ischemic brain injury contribute as significant mechanisms for mortality. Thus, further research opportunities could aim to understand the role in modulation of ASIC1a in astrocytes in order to emphasize potential protective roles in patients who are considered to be “high risk” for stroke and ischemic brain injury. In addition to ASIC1a involvement in neurological diseases, modulation of ASIC1a may reveal a promising therapeutic potential for treatment of gliomas. Since ASIC1a not only plays a significant role in the mechanism of glial tumors, but also in their serious neurological side effects, further research is required to determine how treatments focusing on ASIC1a can mitigate side effects, as well as delay progression of the disease.

### 5.2. Microglial ASICs

Microglia execute the primary role of phagocytosis in the brain [127]. This is important for regular debris clearing in healthy conditions and extensive debris clearing in disease conditions. Physiologically, ASIC1, ASIC2, and ASIC3 are found extensively in microglia [103]. ASIC2 acts primarily as a synergist for ASIC1, helping traffic ASIC1 to the plasma membrane allowing for its normal functioning [106]. Under physiological stimulation, ASIC1 and ASIC2 are upregulated to improve the efficiency of microglial function [103]. ASIC3, on the other hand, is primarily involved in pain sensation and regulation [117]. When microglia do not execute phagocytosis properly, such as when they are overwhelmed during AD, there is prominent plaque build-up. This excess debris accentuates a cytokine storm which contributes to further inflammation and worsens the overall pathological condition [117].

There have been many studies that focus on the relationship between microglial malfunction and ensuing inflammation [103,117]. However, while ASIC1 and ASIC3 are targets during inflammatory states [128], no research directly focuses on the contribution of ASIC1 and/or ASIC3 specifically to the function of microglial cells under physiological or pathological states. It is still unknown how ASICs contribute to the primary role of phagocytosis in microglia, even though the literature shows that these channels are expressed on their membranes. For example, it is known that microglial cells are key players when the brain undergoes liquefactive necrosis, however there is no information in the literature on how microglial ASICs play a role in this condition. Furthermore, while we know that ASIC3 is also expressed in microglial cells and that ASIC3 has a primary function in pain modulation, no studies focus on microglial ASIC3 and how this regulates pain during inflammatory states such as AD. Thus, disruption of each functional ASIC subunit in glial cells is critical for further studies in this field. By focusing on these channel-specific components of microglial cells, targeted therapeutics can be developed which help to address certain aspects of inflammatory diseases [129,130].

### 5.3. Oligodendrocytic ASICs

Oligodendrocytes are myelin-producing cells which insulate axons to allow for enhanced neuronal transmission [110]. ASIC1a is the primary component found in both OPC and mature oligodendrocytes [119]. It has been determined that ASIC1a is utilized in the division and differentiation of OPC through intracellular Ca^2+^ signaling, making it an important component in the myelin formation process [119]. From a pathological standpoint, ASIC1 in oligodendrocytes has been shown to play a major role in neurodegenerative diseases, specifically in MS [123]. Under the acidic conditions of MS, ASIC1a is highly activated in oligodendrocytes. This leads to oligodendrocyte injury and consequent demyelination. Contrarily, when ASIC1 is inhibited by amiloride, myelo-protective and neuroprotective effects are observed [123]. Additionally, ablation of OPC tends to promote obesity [125], whereas inhibition of OPC through antibodies prevents progression of brain cancer [126].

Oligodendrocytic ASIC1 might directly contribute to disease conditions, specifically MS [123]. MS is the primary neurodegenerative disease related to ASIC1 in oligodendrocytes. Future research could concentrate on oligodendrocytic ASIC1 contribution to other demyelinating diseases, such as acute disseminated encephalomyelitis. Moreover, few studies focus on the role of ASIC1 in OPC. ASIC1a in OPC reveals calcium permeability [119], however whether it has a role in leptin signaling needs to be determined; therefore, future studies would need to specifically focus on ASIC1 in OPC in order to confirm its relationship with obesity. In the same regard, OPC proteoglycans are highly expressed in gliomas and definitely contribute to tumor progression [126]. However, no studies focus on the role of ASIC1 in neoplasia. Further studies in the area of glioma therapeutics should target the role of ASIC1 in OPC to determine the consequent impact on glioma growth.

## 6. Conclusions

This review focuses on the theme of ASICs in glial cells from limited literature, summarizing their expression; detailing how ASICs in astrocytes, microglia, and oligodendrocytes contribute to physiological function and how pathological environments perpetuate ASIC dysregulation to result in disease presentations; and describing research gaps between ASICs and glial cells (Table 1). This is important as prior reviews have not gathered ASIC information for each of the glial cell types and discussed how the ASIC-glia relationship relates to specific physiology and pathology.

These findings help organize information for future research to concentrate on channel-specific components in individual glial cells. The gaps outlined in this review provide guidance for areas of concentration in glial ASIC research to promote targeted therapeutic options for various disease conditions.

## Figures and Tables

**Table 1:** ASICs in glial cells: Expression, Function and Research Gap.

Glial Cell Subtype	ASIC Subtype Expression	Physiological Function of Major ASICs	Pathological Focus of ASICs and Research Gap
**Astrocytes**	**1, 2, 3** [77]	**ASIC1a**—influx of Na^+^ and Ca^2+^ ions when pH levels drop below 7.0; rapid depolarization of cell membrane [77,78]. **ASIC2 and ASIC3**—slowly depolarizing to generate sustained currents [77].	ASIC1a activation implicated in neurodegeneration [26,86,87], ischemia [13,92,93], and glioma [97,98]. ***Research Gap:*** explore the role of ASIC1a in astrocytes towards neurodegenerative diseases, ischemia, and cancer. ***Research Gap:*** developing a more detailed understanding of the role of ASIC2 and ASIC3 in astrocytes in pathological processes.
**Microglia**	**1a, 2a, 3** [103]	**ASIC1a**—influx of Na^+^ and Ca^2+^ ions when pH levels drop below 7.0; rapid depolarization of cell membrane [14,103]. **ASIC2a**—activated when pH levels drop below 5.5; increases ASIC1 co-localization [107,108]. **ASIC3**—activated when pH levels drop below 7.0; pain modulation [15,21].	ASIC1a and ASIC2a overexpression in plaque accumulation of Alzheimer’s Disease (AD) [86,103,116]. ***Research Gap:*** targeted research on microglial ASIC1a and ASIC2a in AD. ASIC3 overexpression in hyperalgesia [87]. ***Research Gap:*** specific understanding of microglial ASIC3 in pain modulation.
**Oligodendrocytes**	**1a, 2a, 4** [118,119]	**ASIC1a**—influx of Na^+^ and Ca^2+^ ions when pH levels drop below 7.0; Rapid depolarization of cell membrane [119]. **ASIC2a**—modulation of ASIC1a surface expression [107,108]. **ASIC4**—***Research Gap***	ASIC1a activation implicated in multiple sclerosis (MS) [122]. ***Research Gap*:** understanding the specific role of oligodendrocyte ASIC1 in MS. ASIC2 knockout associated with better clinical scoring in EAE. ***Research Gap:*** exploring oligodendrocyte ASIC2 in MS. ***Research Gap:*** understanding oligodendrocyte ASIC4 in disease.

## Data Availability

Not applicable.

## Abbreviations

AD	Alzheimer’s disease
ASICs	acid-sensing ion channels
CNS	central nervous system
DEG	degenerin
DTA	diphtheria toxin A
EAE	experimental autoimmune encephalitis
ENaC	epithelial sodium channel
GFAP	glial fibrillary acidic protein
GMQ	2-guanidine-4-methylquinazoline
H_V_	voltage-gated proton channel
HSV-TK	herpes simplex virus-thymidine kinase
KO	Knock-out
LPS	lipopolysaccharides
LTD	long-term depression
LTP	Long-term potentiation
MCAO	middle cerebral artery occlusion
MS	multiple sclerosis
NO	nitric oxide
OLC	oligodendrocyte lineage cells
OPC	oliodendrocyte precursor cells
PcTx1	psalmotoxin-1
PD	Parkinson’s disease
pH_50_	half activation value of ASICs by pH
ROS	reactive oxidative species
WT	wild-type
